# A novel loss-of-function variant in *STAT1* causes Mendelian susceptibility to mycobacterial disease

**DOI:** 10.3389/fcimb.2025.1595389

**Published:** 2025-05-26

**Authors:** Kunlun Lv, Zhuoqing Gong, Yiting Fu, Sisi Zhao, Yinggai Song, Huijun Wang, Zhimiao Lin

**Affiliations:** ^1^ Dermatology Hospital, Southern Medical University, Guangzhou, China; ^2^ Department of Dermatology, Peking University First Hospital, Beijing Key Laboratory of Molecular Diagnosis on Dermatoses, National Clinical Research Center for Skin and Immune Diseases, Beijing, China

**Keywords:** STAT1, Mendelian susceptibility to mycobacterial disease, loss-of function, gene variant, *Mycobacterium marinum*

## Abstract

**Introduction:**

Mendelian Susceptibility to mycobacterial disease (MSMD) is a rare inherited immunodeficiency disorder characterized by increased susceptibility to atypical mycobacterial infections induced by defective IFN-γ pathway.

**Methods:**

We report three patients from a family presenting with multiple osteolytic lesions and cutaneous granulomas due to Mycobacterium marinum infections. Functional studies, including Western blotting and immunofluorescence, assessed phosphorylation and nuclear translocation of the mutant STAT1-Ile707Thr in eukaryotic overexpression systems. A luciferase reporter assay evaluated its transcriptional activity. Additionally, structural analysis using AlphaFold3 predicted the variant’s functional impact.

**Results:**

A novel STAT1 variant (c.2120T>C, p.Ile707Thr) was identified. The STAT1-Ile707Thr mutant exhibited reduced phosphorylation and impaired nuclear translocation compared to wild-type STAT1. The luciferase assay confirmed decreased transcriptional activity. AlphaFold3-based cluster analysis supported a loss-of-function effect of the mutant.

**Discussion:**

This study expands the spectrum of STAT1 variants and microbial pathogens associated with MSMD.

## Introduction

Mendelian susceptibility to mycobacterial disease (MSMD, OMIM: 209950) is a group of inborn error of immunity (IEI) with genetic heterogeneity characterized by a selective predisposition to weakly virulent mycobacteria infection such as environmental mycobacteria and BCG vaccine (Noma et al., 2022). Most MSMD patients are early-onset, and adult-onset MSMD has also been occasionally recorded (Ye et al., 2022; Bustamante et al., 2014). At least 38 genetic etiologies have been identified to be associated with MSMD (Casanova et al., 2024), among which *STAT1*, *IFNGR1*, *IFNGR2, IRF1*, and *IL12RB1* play critical roles in the IFN-γ-mediated immunity pathway, as illustrated in **Supplementary Figure S1** (Casanova et al., 2024). The prevalence of MSMD is approximately 1/50,000 (Noma et al., 2022).

The *STAT1* gene encodes the signal transducer and activator of transcription 1 (STAT1), a transcription factor of the STAT family composed of seven domains, including the SH2 domain and the C-terminal transactivation domain (TAD) (Hu et al., 2021). STAT1 is activated by various cytokines, notably IFN-α/β and IFN-γ (Boisson-Dupuis et al., 2012). IFN-α/β is involved in antiviral immunity, while IFN-γ contributes to antimicrobial immunity (Hu et al., 2021; Boisson-Dupuis et al., 2012; Rosain et al., 2023). Upon the stimulation with IFN-γ, STAT1 is phosphorylated at the Tyr701 residue, followed by the formation of the scissor-like homodimers, named IFN-γ activation factor (GAF). GAF is stabilized by the interaction between the SH2 domain of one monomer and the p-Tyr701 of the other one. After nuclear translocation, GAF binds to gamma-activating sequences (GAS) in the promoter region of downstream IFN-stimulated genes and regulates their expression levels, including transcription factors IRF1 and IRF8, both of which promote the expression of IL-12 and IL-23 (Casanova et al., 2024; Kagawa et al., 2017; Marg et al., 2004). Particularly, IL-23 plays a significant role in IFN-γ-mediated anti-mycobacterial immunity (Philippot et al., 2023). The STAT1-Y701C variant that is located at the phosphorylation site of STAT1 is one of the typical loss-of-function variants associated with MSMD (Dupuis et al., 2001). Monoallelic loss-of-function variants in *STAT1* cause isolated MSMD, while biallelic loss-of-function variants in *STAT1* lead to syndromic MSMD, clinically characterized by concurrent mycobacterial infections with other pathogens encompassing viruses, bacteria, and fungi, demonstrating potential lethality in clinical cases (Ye et al., 2022; Casanova et al., 2024; Kagawa et al., 2017).

Herein, we report three patients from a family presented multiple osteolytic lesions and cutaneous granulomas due to *Mycobacterium marinum* infection. A underlying heterozygous variant in *STAT1* (c.2120T>C, p.Ile707Thr) was identified, which was near the phosphorylation site and demonstrated to exert a loss-of-function property.

## Materials and methods

### Individuals

This study was approved by the Clinical Research Ethics Committee of the Southern Medical University and was conducted following the principles of the Declaration of Helsinki. A family with three patients was enrolled in this study. We obtained written informed consent from the individuals or their guardians prior to sample collection and publication of photographs.

### Genomic DNA extraction and real-time PCR

Skin tissues obtained from the left forearm and right upper arm of I-1 were formalin-fixed and paraffin-embedded (FFPE). Genomic DNA was extracted from sectioned FFPE (10 μm thickness) using the QIAamp DNA FFPE Tissue kit (56404, QIAGEN, Germany) according to the manufacturer’s instructions. For pathogen detection, extracted DNA served as the template in real-time PCR conducted with specific primers and probes targeting a group of *Mycobacterium* (including *Mycobacterium tuberculosis* and nontuberculous mycobacteria) and 14 common *Mycobacterium* species. Details were described previously (Song et al., 2022; Xiao et al., 2024).

### Genetic analysis

Whole-exome sequencing (WES) was carried out using genomic DNA extracted from peripheral blood samples of II-2 at MyGenostics (Beijing, China) as described previously (Wang et al., 2020). Sanger sequencing was performed on three patients and their unaffected family members to verify the WES results and co-segregation using primers for exon 23 of *STAT1* (forwards: GGCTAAGCTGTCTAGAAACAG and reverse: CTCAACAAGTTCAGCTGTGA).

### Histological analysis

Skin tissue obtained from the left thigh of I-1 and the chest of II-2 were fixed with 4% paraformaldehyde, embedded in paraffin, and sectioned (3μm thickness). The slides were subjected to hematoxylin-eosin (HE) staining for general morphology, acid-fast staining for Mycobacterium detection, and immunofluorescence using anti-p-STAT1 antibody (Cell Signaling Technology, Cat# 9167, RRID: AB_561284).

### Modeling and prediction

Predicted 3D models of the wild-type and the STAT1-Ile707Thr variant were acquired by AlphaFold3 online service and visualized using PyMOL software. The crystal structures (PDB code 1BF5 for the phosphorylated dimer, and PDB code 6TLC for the unphosphorylated dimer) were employed as templates. The analysis was conducted using Python 3.7 with Biopython, the associated code is publicly available in the GitHub repository at https://github.com/MDhewei/Bioinforbricklayer (He, 2023).

### Plasmid constructions

Full-length *STAT1* (GenBank: NM_007315.4) open reading frame was amplified and cloned into pCMV-MCS-3XFlag-SV40 vector containing a C-terminal FLAG tag. Mut Express II Fast Mutagenesis Kit (C214, Vazyme, China) was used to create the plasmids expressing the STAT1-Ile707Thr variant, with appropriate primers (forwards: CTGAGTTGACTTCTGTGTCTGAAGTTCACCCTT and reverse: CACAGAAGTCAACTCAGTCTTGATATATCCAGTTCC).

### Cell culture, transfection, and stimulation

HEK293T cells were cultured in DMEM (11965126, Gibco) supplemented with 10% FBS (A5670701, Gibco) at 5% CO_2_ and 37°C. Expression plasmids were transiently transfected into HEK293T cells using Lipofectamine 3000 (L300001, ThermoFisher). IFN-γ (10^5^ IU/mL, C014, Novoprotein, Suzhou, China) was added to stimulate the cells for either 30 or 60 minutes, with phosphate buffer saline (PBS) serving as control.

### Protein extraction and western blotting

Cells were lysed using IP lysis buffer (87788, ThermoFisher) containing protease inhibitor cocktail (04693132001, Merck) and protein phosphatase inhibitors (FD1002, Fudebio-tech, Hangzhou, China). The protein samples were separated by NuPAGE™ 4-12% Bis-Tris Protein Gels (NP0322BOX, ThermoFisher) and transferred onto NC membranes. The blots were blocked with 5% milk at room temperature for 1 h, followed by overnight incubation at 4°C with primary antibodies, including GAPDH (ZSGB-Bio Cat# TA-08, RRID: AB_2747414), FLAG (Sigma-Aldrich Cat# F1804, RRID: AB_262044), and STAT1 (Abcam Cat# ab109457, RRID: AB_10865748). After the incubation with secondary antibodies, immunoreactivity was detected using Immobilon Western Chemiluminescent HRP Substrate (WBKLS0500, Millipore).

### Immunofluorescence

After 24 hrs of culture, transfected HEK293T cells in chamber slides were stimulated with IFN-γ (10^4^ IU/mL) or PBS for 30 minutes. Cells were then fixed with paraformaldehyde, permeabilized with 0.1% Triton, and blocked with goat serum for 30 minutes. After the overnight incubation with anti-FLAG (Sigma-Aldrich Cat# F1804, RRID: AB_262044) at 4°C, cells were incubated with the corresponding fluorescein-conjugated secondary antibody at room temperature for 1h. Images were captured using a laser confocal microscope (Nikon, A1R-HD25) after being mounted with DAPI (ZLI-9557, ZSGB-BIO).

### Luciferase reporter assay

HEK293T cells cultured in 24-well plates were transfected with pGAS (HanYi Biosciences Inc.), Renilla (HanYi Biosciences Inc.) and plasmids expressing wild-type STAT1 or STAT1-Ile707Thr variant, with a ratio of 200:2:300. The relative luciferase units (RLUs) were measured on above cells stimulated with IFN-γ (10^4^ IU/mL) or PBS for 24 hrs, according to the manufacturer’s instructions. The RLUs were normalized with the Renilla signal and analyzed using ANOVA with GraphPad Prism 8.

## Results

### Clinical features of patients

**Figure 1:**
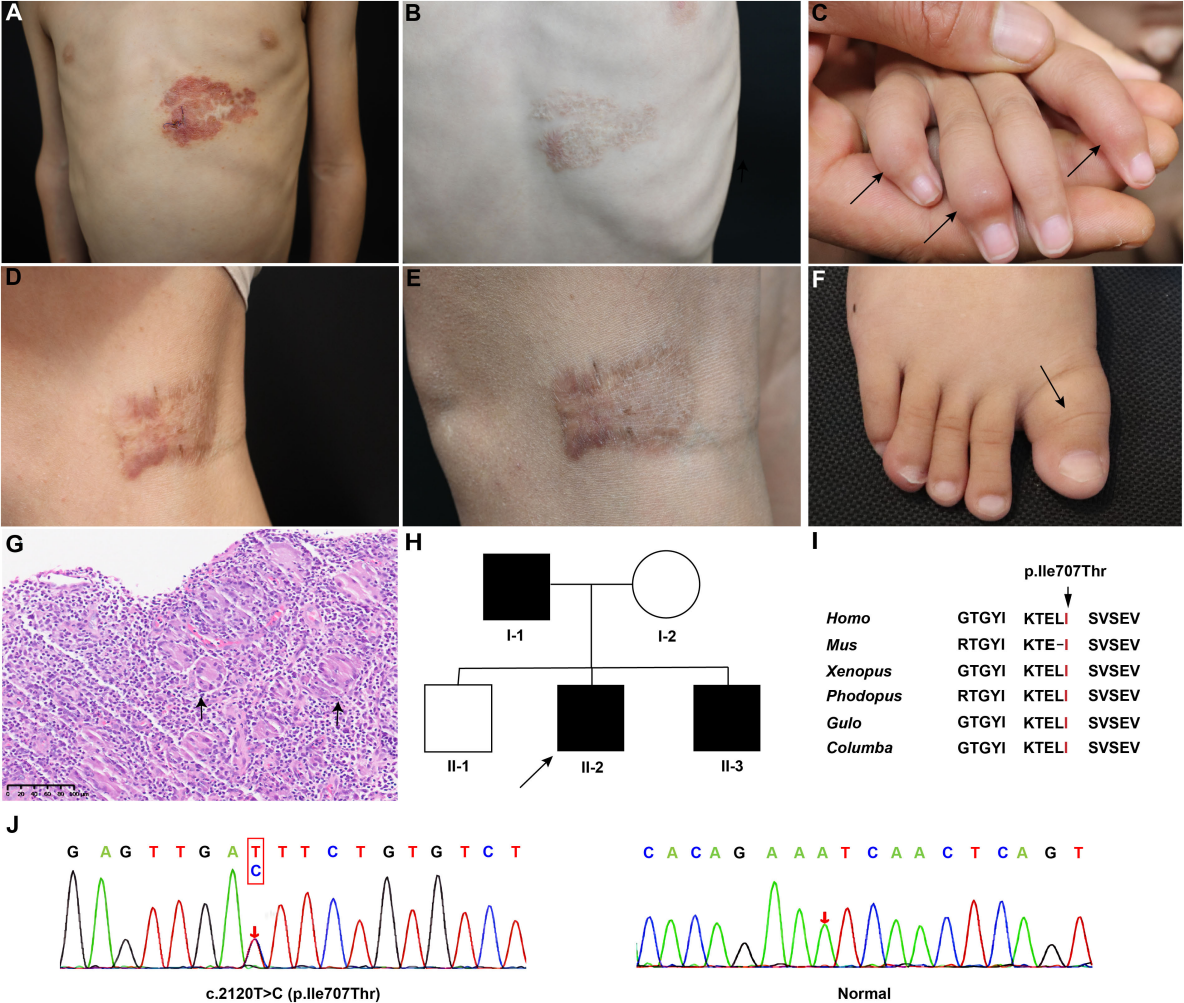
Yellow-reddish plaque on the left chest of II-2 before **(A)** and after **(B)** treatment. Swollen enlargements of phalanges in (the 2^nd^, 4^th^, and 5^th^) digits of the right hand **(C)** and the right first toe **(F)** of II-3 (indicated by triangular symbols). Reddish plaque on the inside of right knee of I-1 before **(D)** and after **(E)** treatment. **(G)** Hematoxylin and eosin staining of II-2’ skin lesion showed the presence of inflammatory granulomas and a significant abundance of multinucleated giant cells. **(H)** Pedigree of the family. The arrow indicated the proband. **(I)** Residue Isoleucine 707 is highly conserved among species. **(J)** The pathogenic variant of *STAT1*. The arrow indicates the variant.

Three patients from a family with MSMD were recruited, with clinical features summarized in **Table 1**. II-2, a 4-year-old boy, presented with hypertrophy of the right first metatarsal bone at 2 years old, and the bone enlargement progressively increased. He also presented with progressive papules on the trunk. Over the past two years, he has experienced recurrent fever for more than two years, with a maximum body temperature of 39 °C, accompanied by cough and white sputum, which was diagnosed with pneumonia in the local hospital. Physical examination revealed a yellow-reddish lobulated plaque on his left chest, approximately 10*8 cm, surrounded by yellow-reddish papules (**Figure 1A**). The plaque’s surface tended to bleed when touched. The right elbow and knee joints of II-2 were also enlarged without redness or swelling, leading to limited mobility. Multiple enlarged cervical lymph nodes and axillary lymph nodes were observed. X-ray imaging revealed bone destruction with low bone density in limbs. Histopathological analysis of the skin lesion suggested necrotizing granulomatous inflammation (**Figure 1G**). Acid-fast staining was positive. II-3, the younger brother of II-2, presented with similar symptoms, including progressive limb bone enlargement, enlarged lymph nodes, and papules on the trunk. He also experienced recurrent fever and pneumonia, which responded to anti-infective therapy. Notably, he had enlargements of phalanges in the 2^nd^, 4^th^, and 5^th^ digits of the right hand (**Figure 1C**), the right first toe (**Figure 1F**), and the left metatarsals. X-ray imaging of his hands and feet revealed bone destruction with low bone density. I-1, father of II-3 and II-2, initially developed erythematous plaques on the right ankle at the age of 26. The plaques progressed to involve the faciocervical regions, limbs, and the medial aspect of the right knee (**Figure 1D**). He denied the history of frequent respiratory or other infections. Though conventional microbiological culture or metagenomics sequencing failed to identify pathogens in II-2 and I-1, *Mycobacterium marinum* was detected in the FFPE tissue of I-1 by real⁃time PCR. Thereafter, clarithromycin (0.125g) twice daily and rifampicin (0.15g) once daily were administered to three patients. After six months of regular treatment, substantial alleviation of the skin lesions was observed in both patients (**Figure 1B, E**). The enlarged bones of II-2 and III-3 were also recovered.

**Table 1:** Clinical findings in patients.

Patient	II-2	II-3	I-1
**Age at clinical examination**	4-year-old	2-year-old	31-year-old
**Sex**	male	male	male
**Weight**	13.6 kg	10.1 kg	61.0 kg
**Height**	98.0 cm	83.1 cm	171.0 cm
**Temperature**	36.6 °C	36.5 °C	37.0 °C
**Heart rate**	78 bpm	98 bpm	85 bpm
Clinical features			
Lymph node	Multiple lymph node enlargements occurred in the neck, armpit, and pulley.	–	–
Bone	Enlargement in the right toes and right index finger bones; Swelling in the right elbow and knee joints.	Enlargements in the right first toe, left metatarsal, and the first, fourth, and fifth phalanges of the left hand; The neck was rigid, with restricted movement.	–
Laboratory testing:	CRP: 91mg/L↑; IFN-γ:7.07pg/ml↑; IL-6: 76.17↑; IL-10: 0.48pg/ml; TNF-α: 1.83pg/ml; T-spot test (-), mycobacterial nucleic acid test (-); mycobacterial culture (-); PPD test: 72-hours (+++);	WBC:14.75*10^9↑; RBC;3.53*10^12↓; CRP: 70.11mg/L↑; PPD test: 72-hour (+++); T-spot (-);	WBC: 10.78*10^9↑; CRP88.66mg/L ↑; PCR test for Mycobacterium antigen (-); T-spot (+); PDD: 48h (+), 72h (-);
CT:	A small patch of slightly high density in the posterior basal segment of the right lower lobe and enlarged lymph nodes in both axillae were visible.	A small amount of fibrinous strands in the lingual segment of the left upper lobe.	Fibrotic lesions was observed in the lingular segment of the left lung, with adhesions to the anterior pleura.
X-ray:	Multiple areas of low-density bone destruction were observed in limbs.	Multiple low-density areas in limbs were observed.	–
Special staining	D-PAS (-), Gram stain (-), and Acid-Fast (+).	–	PAS (-); Acid-Fast (-).
treatments	Oral administration of clarithromycin (0.125g) twice daily and rifampicin (0.15g) once daily.		
Outcomes	The condition of those patients improved, and the plaques nearly disappeared.		

Absent, -; bpm, beats per minute; CT, Computer Tomography; WBC, White blood cell; RBC, red blood cell; CRP, C-reactive protein; PPD, pure protein derivatives.

### Identification and bioinformatic analysis of the variant in *STAT1*

To identify the underlying genetic defect, WES was performed on II-2. A heterozygous variant c.2120T>C (p. Ile707Thr) in *STAT1* (Genbank: NM_007315.4) was detected (**Figure 1J**). Sanger sequencing verified the variant in the proband and confirmed the co-segregation of the variant with the phenotype in the family. The Ile707 is highly conserved across different species (**Figure 1I**), and the variant is absent in all known public databases. The pedigree chart is shown in **Figure 1H**.

**Figure 2:**
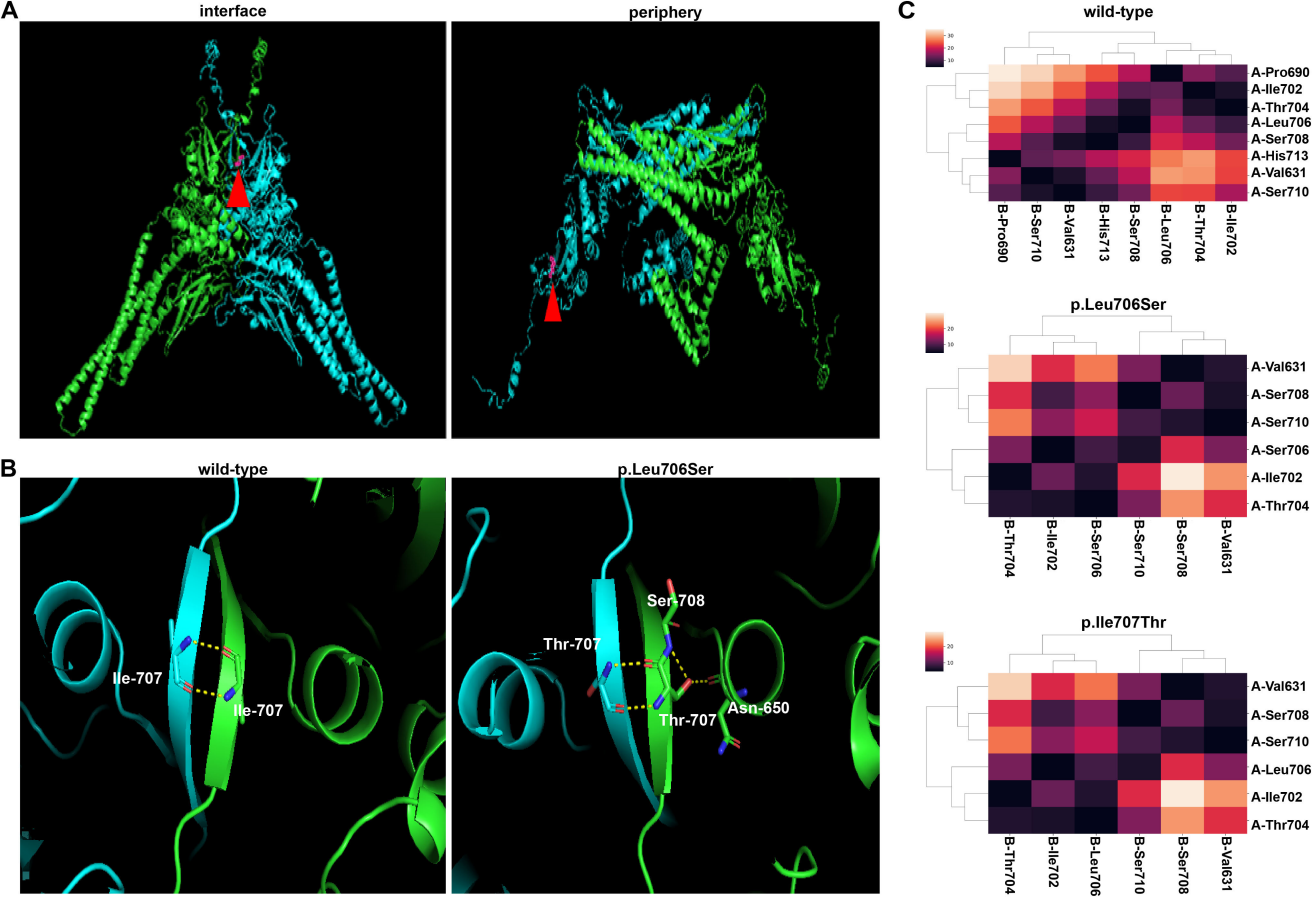
Modeling and prediction of the STAT1 dimer. **(A)** Ile707 of STAT1 is positioned at the interface of the phosphorylated dimer and the periphery of the unphosphorylated dimer. **(B)** The presence of hydrogen bonds in AlphaFold3-predicted structures of STAT1 dimers (only showing the hydrogen bonds in Chain A). In the STAT1^wild-type^-STAT1^wild-type^ dimer, hydrogen bonds exist between residues Ile707 in Chain A and **(B)** While in the STAT1^Ile707Thr^-STAT1^Ile707Thr^ dimer, Chain A shows new hydrogen bonds linking Thr707 to Ser708 and Asn 650 in monomer, the same changes also appear in Chain B (not shown in this panel). **(C)** The cluster analysis of the interface between STAT1 phosphorylated homodimer of wild-type (the upper panel), STAT1-Ile707Thr (the middle panel), STAT1-Leu706Ser (the lower panel). Two chains of the dimer were designated as A (blue) and B (green). The threshold was set at 5 **(Å)** Residues involved in the phosphorylated dimer formation were included in the heatmap. STAT1-Ile707Thr and STAT1-Leu706Ser showed decreased residues in the interface of phosphorylated dimer.

Structural analysis of the AlphaFold3-predicted structure of STAT1 revealed distinct spatial position of the residue Ile707 in phosphorylated and unphosphorylated STAT1 homodimers. In the phosphorylated state, the residue Ile707 is located at the interface of the homodimer, while at the periphery in the unphosphorylated state, suggesting the residue Ile707 might involve in the formation of phosphorylated homodimer (**Figure 2A**). Structural alteration of the phosphorylated homodimer induced by the STAT1-Ile707Thr variant was revealed by the AlphaFold3-predicted structure. Although the number of hydrogen bonds increased when Ile707 was replaced by residue Threonine, those excessive hydrogen bonds may disrupt the native hydrophobic environment inside of protein, ultimately destabilize the dimer (**Figure 2B**). As shown in **Figure 2C**, cluster analysis of inter-monomeric contacts in wild-type phosphorylated dimers (designated as Chain A and Chain B) illustrated critical contribution of residues 631 to 710 located at the SH2 domain and TAD domains, consistent with previous findings (Boisson-Dupuis et al., 2012). However, the Ile707Thr variant exhibited substantial reduction of interaction residues in Chain A’s SH2 domain (**Figure 2C**), mirroring the interfacial defect observed in the established loss-of-function variant, STAT1-Leu706Ser (Dupuis et al., 2003). These computational evidences collectively suggest that the Ile707Thr variant might impair the function of STAT1 via interfering with the structure and stability of the phosphorylated dimer.

### The STAT1-Ile707Thr variant led to a lower response to IFN-γ and reduced GAS activity

**Figure 3:**
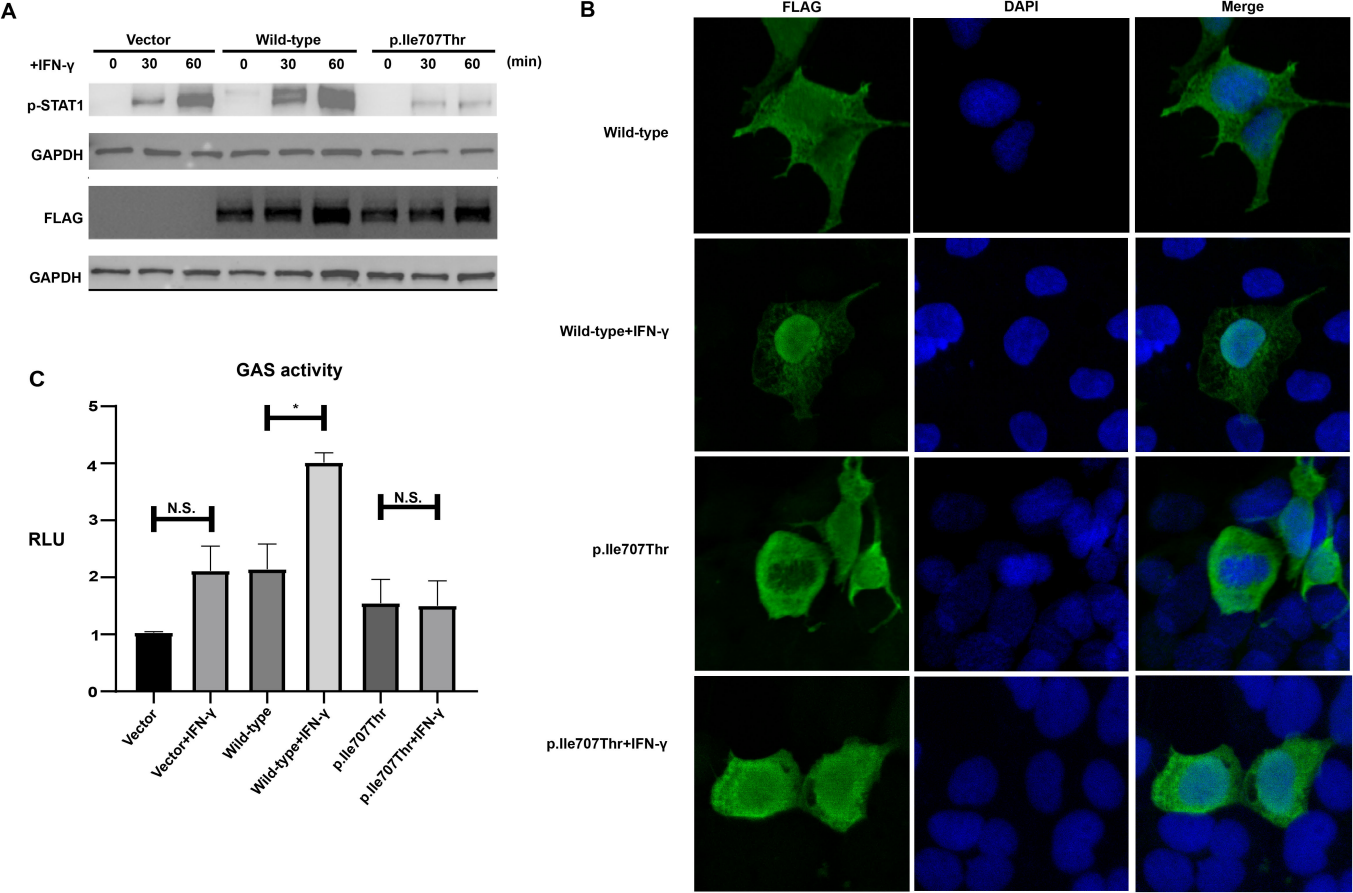
Impaired phosphorylation, subcellular localization and transcriptional activity of STAT1-Ile707Thr following IFN-γ stimulation. **(A)** Western blot analysis of FLAG-tagged total STAT1 and p-STAT1 from HEK293 T cells expressing wild-type and the STAT1-Ile707Thr variant. GAPDH was used as a loading control. A comparable level of total STAT1 was observed in cells expressing wild-type and the STAT1-Ile707Thr variant. Upon stimulation with IFN-γ, a distinct band corresponding to p-STAT1 was observed in the cells expressing wild-type and empty vector, while only a faint band corresponding to p-STAT1 was detected in cells expressing STAT1-Ile707Thr variant. **(B)** Subcellular localization of STAT1. Immunofluorescence staining for FLAG-tagged STAT1 in cells expressing wild-type and the STAT1-Ile707Thr variant. Both wild-type and STAT1-Ile707Thr variant localized in the cytoplasm and nucleus without any stimulation. After a 30-minute IFN-γ stimulation, the wild-type STAT1 accumulated in the cellular nucleus, while the STAT1-Ile707Thr variant still distributed in the cytoplasm and nucleus. Scale bar: 20μm. **(C)** Luciferase assay of GAS-induced activity in HEK 293T cells transfected with FLAG-tagged STAT1, pGAS, and Renilla. The RLUs were normalized using the Renilla signal. After stimulation with IFN-γ for 24 hrs, a substantial increase of transcriptional activity was observed in cells expressing the wild-type STAT1, while no significantly increased transcriptional activity was detected in cells expressing the STAT1-Ile707Thr variant. Error bars represent the SEM of three independent experiments. *p < 0.05. n.s. means no significance.

To investigate the phosphorylation level of the STAT1-Ile707Thr variant, Western blot analysis was performed on HEK293T cells transfected with wild-type or the STAT1-Ile707Thr variant. The expression level of total STAT1 was comparable in cells expressing the wild-type and the STAT1-Ile707Thr before and immediately after the stimulation of IFN-γ (**Figure 3A**). Intriguingly, after 30 and 60 minutes of IFN-γ stimulation, there was a significant reduction of the phosphorylated STAT1 in cells expressing the STAT1-Ile707Thr variant, compared to those expressing the wild-type STAT1 (**Figure 3A**), suggesting the impaired phosphorylation of the STAT1-Ile707Thr variant.

IF was performed on transfected HEK293T cells using the antibody against the FLAG tag to assess the subsequent nuclear translocation. Before the IFN-γ stimulation, both wild-type STAT1 and the STAT1-Ile707Thr variant were distributed in the cytoplasm and nucleus (**Figure 3B**). After 30 minutes of IFN-γ stimulation, the wild-type STAT1 concentrated in the nucleus, while the distribution of STAT1-Ile707Thr variant showed no discernible difference (**Figure 3B**), demonstrating the blocked nuclear translocation of the STAT1-Ile707Thr variant. Consistently, IF of p-STAT1 in the FFPE tissue showed significantly decreased p-STAT1 expression and reduced nuclear localization of STAT1 in I-1 (**Supplementary Figure S2**).

To explore whether the variant suppressed the transcriptional activity of GAS, a luciferase reporter assay was carried out on transfected cells. Upon the stimulation of IFN-γ, the wild-type STAT1 displayed a significant increase in transcriptional activity, while only a slight increase in transcriptional activity was induced by the STAT1-Ile707Thr variant (**Figure 3C**). These findings suggested that the STAT1-Il707Thr variant exhibits a loss-of-function effect, resulting in the inhibition of IFN-γ signaling.

## Discussion

Variants in *STAT1* can induce susceptibility to different pathogens by different pathogenic effects. Autosomal-dominant (AD) gain-of-function (GOF) variants in *STAT1* result in chronic mucocutaneous candidiasis (CMC) (Hartono et al., 2018), while AD *STAT1* deficiency that impairs type II IFN-induced STAT1-mediated signaling could lead to isolated MSMD (Casanova et al., 2024). Autosomal-recessive (AR) *STAT1* deficiency disrupts both type I and type II IFN-induced STAT1-mediated signaling, resulting in syndromic MSMD (Majoros et al., 2017). The mycobacterial infection, especially caused by virulent mycobacteria was common in patients with variants in *STAT1*, suggesting a significant role of STAT1 in the immune response to mycobacterial infections (Rosain et al., 2023). Herein, we identified three patients in a family suffering from MSMD who harbored a novel heterozygous variant in *STAT1* (c.2120T>C, p.Ile22Thr). *In silico* analysis and *in vitro* experiments revealed that the STAT1-Ile707Thr variant exhibited a loss-of-function effect via diminished phosphorylation and impaired nuclear entry.

The patients’ presentation of multiple osteolytic lesions with recurrent fever was similar to chronic recurrent multifocal osteomyelitis (CRMO), a noninfectious autoinflammatory disorder characterized by multifocal and recurrent bone inflammation (Hofmann et al., 2017). Although CRMO diagnosis remains challenging due to the lack of validated biomarkers, decreased IL-10 levels might be a clue (Hofmann et al., 2017). This phenomenon was not observed in patient II-2, whose IL-10 level was within the normal range (details in **Table 1**). Besides, osteomyelitis is a common concurrent symptom in MSMD patients with AD *STAT1* deficiency, potentially caused by impaired-IFN-γ mediated suppression of osteoblast differentiation and bone resorption (Noma et al., 2022; Hirata et al., 2013; Zhang et al., 2021). Considering *Mycobacterium marinum* identified by real-time PCR in the lesion of I-1, and significant improvement after the antimycobacterial therapy, the diagnosis of mycobacterial osteomyelitis was confirmed rather than autoinflammatory disorder.

*Mycobacterium marinum* belongs to photochromogenic Group I non-tuberculous Mycobacteria according to Runyon’s classification (Hashish et al., 2018). It was reported that *Mycobacterium marinum* infection characteristically presents with nodular cutaneous lesions that could progress to osteomyelitis and arthritis, which were presented in our patients (Aubry et al., 2002). Additionally, outdoor swimming exposure was documented in subject I-1, which aligns with prior studies identifying contaminated water as the confirmed infection source (Tsai et al., 2007). No similar exposure was reported in II-2 and II-3, while potential environmental exposure factors could not be excluded. Though nontuberculous mycobacteria (NTM) are predominantly environmental pathogens, with rare documented cases of person-to-person transmission (Bryant et al., 2013), a case of interpersonal transmission of *Mycobacterium abscessus* in immunodeficiency patients has been reported previously (Bryant et al., 2013). Three patients herein with a deficiency in antimycobacterial immunity had prolonged cohabitation, raising the possibility of person-to-person transmission. Besides, clarithromycin and rifampin have been proven effective in the treatment of NTM infections, including *Mycobacterium marinum*, as described in previous studies (Tsai et al., 2007). Therefore, we hypothesized that II-2 and II-3 were probably infected by *Mycobacterium marinum*, although direct evidences of the pathogen were lacking.

Notably, despite MSMD patients’ susceptibility to *Mycobacterium avium* complex (MAC) infections (except tuberculosis mycobacterial infections), *Mycobacterium marinum* has not been previously reported in MSMD or *STAT1* LOF patients (**Supplementary Table S1**) (Ye et al., 2022; Frieß et al., 2024; Chen et al., 2022; Bryant et al., 2013; Khavandegar et al., 2024). This finding could be explained by the low reported incidence of *Mycobacterium marinum* (0.04/100,000 annually in France), compared to the epidemiological predominance of MAC in nontuberculous mycobacterial (Matsuyama et al., 2016; Khavandegar et al., 2024; Aubry et al., 2002). Further large-scale prospective studies are required to determine the prevalence of *Mycobacterium marinum* infections among MSMD patients. Our findings underscore the pivotal importance of accurate pathogen identification, particularly given that *Mycobacterium marinum* exhibits heightened resistance to several widely utilized antimycobacterial agents, such as streptomycin, isoniazid, and pyrazinamide (Tsai et al., 2007). This resistance complicates treatment, as standard antimycobacterial treatment may be ineffective.

Previously, Kagawa et al. demonstrated that most GOF variants of *STAT1* are located in the interface of unphosphorylated STAT1 dimer, while variants acting as LOF are more frequently located at the interior region of phosphorylated dimer (Kagawa et al., 2017). Given that the Ile707 residue is located at the periphery of the unphosphorylated dimer and the interface of the phosphorylated dimer, it is plausible to infer that the STAT1-Ile707Thr variant induces the dysfunction of the phosphorylated dimer. AlphaFold3 accurately predicted the structure of the phosphorylated dimer, and subsequent cluster analysis of the results revealed a reduction in the number of residues involved in dimer formation for both the STAT1-Ile707Thr variant and a previously reported loss-of-function (LOF) variant, STAT1-Ile706Ser variant (Dupuis et al., 2003). Consistently, our *in vitro* analysis demonstrated that the STAT1-Ile707Thr variant impairs phosphorylation, translocation, and GAS transcription activity of STAT1 dimers.

## Data Availability

WES data is available upon request.
