# Supplementary material for: A novel loss-of-function variant in STAT1 causes Mendelian susceptibility to mycobacterial disease
Source: Front Cell Infect Microbiol. 2025 May 26;15:1595389. doi: 10.3389/fcimb.2025.1595389 (PMC12146302; doi:10.3389/fcimb.2025.1595389)
Supplement: Supplementary file 1 [file DataSheet1.pdf]

Supplementary Material

Supplementary Table S1. Pathogen spectrum of *STAT1* deficiency patients.

Pathogen Spectrum	All Patients (n=70)	AD STAT1 deficiency (n=36)	AR STAT1 deficiency (n=34)
M. bovis-BCG	34 (48.6%)	22 (61.1%)	12 (35.3%)
Mycobacterium avium	13 (18.6%)	4 (11.1%)	9 (26.5%)
Mycobacterium tuberculosis	8 (11.4%)	8 (22.2%)	0
Mycobacterium kansasii	2 (2.9%)	0	2 (5.9%)
Mycobacterium abscessus	1 (1.4%)	0	1 (2.9%)
Mycobacterium szulgai	1 (1.4%)	0	1 (2.9%)
Mycobacterium scrofulaceum	1 (1.4%)	0	1 (2.9%)
Mycobacterium marinum	1 (1.4%)	1 (2.8%)	0
Mycobacterium malmoense	1 (1.4%)	0	1 (2.9%)
Undefined species	3 (4.3%)	1 (2.8%)	2 (5.9%)
Viral infections	25 (35.7%)	5 (13.9%)	20 (58.8%)
Other bacterial infections	11 (15.7%)	2 (5.6%)	9 (26.5%)
Fungal Infection	8 (11.4%)	0	8 (23.5%)

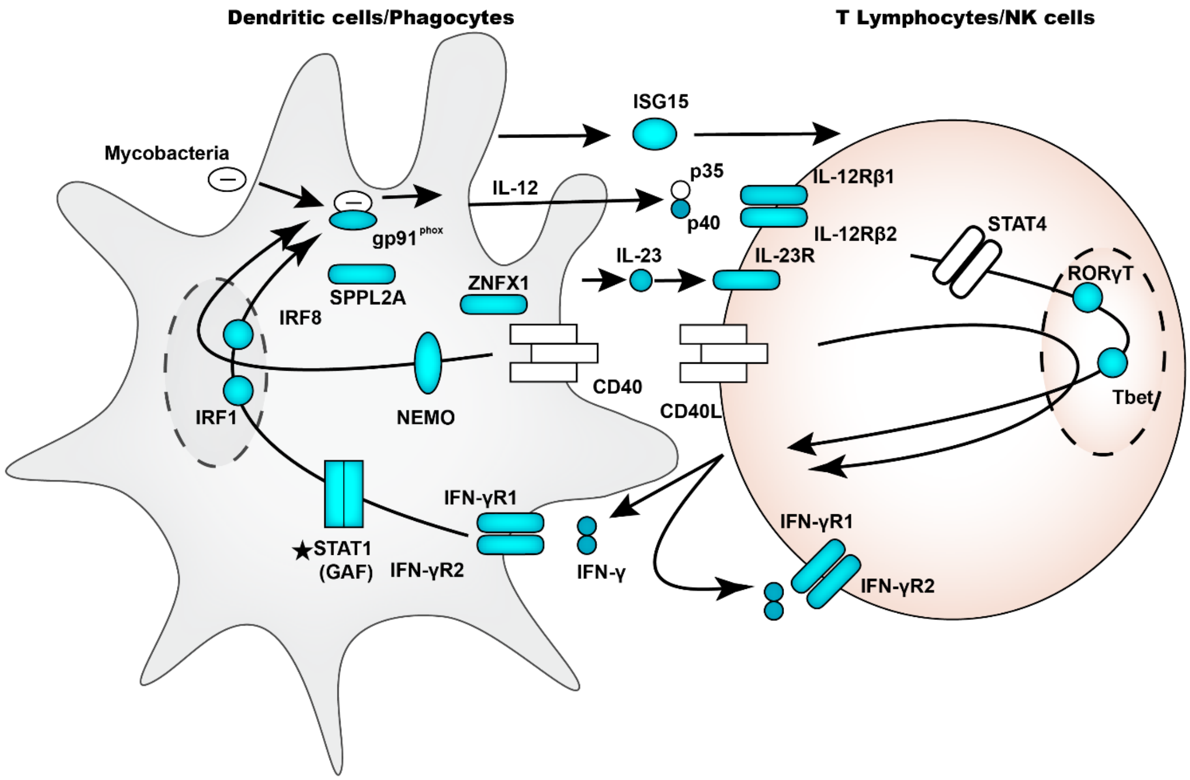

Supplementary Figure S1. Proteins involved in the IFN-γ pathway during Mycobacteria infection. Proteins for which mutations in the corresponding genes have been identified to cause Mendelian susceptibility to mycobacterial disease (MSMD) are indicated in blue. The star indicates the protein of interest in our study (STAT1). ZNFX1, NFX1-type zinc finger-containing protein 1; SPPL2A, signal peptide peptidase-like 2A; NEMO, NF-kB essential modulator. GAF, g-activating factor.

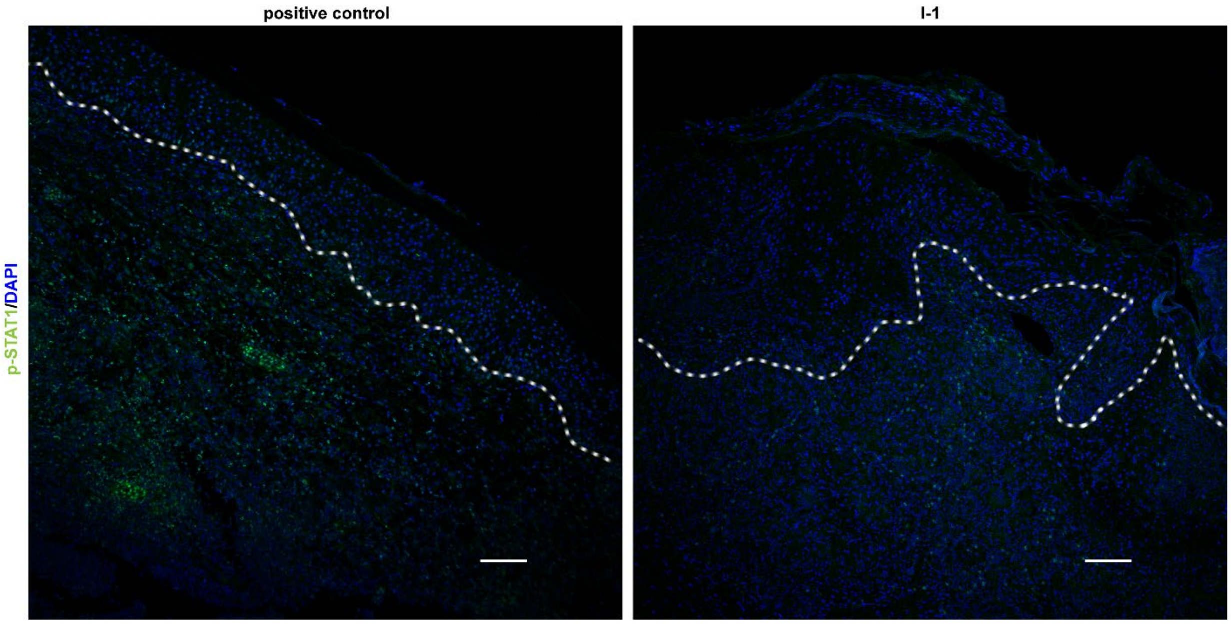

Supplementary Figure S2. Expression of p-STAT1 in lesional tissue of patient I-1. Immunofluorescence staining for p-STAT1 in the lesional tissue of I-1 and a positive control (skin tissues of cutaneous tuberculosis). In both I-1 and the positive control, p-STAT1 exhibits nuclear and perinuclear localization. Compared with the positive control, markedly reduced p-STAT1 expression and less nuclear accumulation were detected in the skin tissue of patient I-1. The white dotted line represents the border between the epidermis and dermis. Scale bar: 100μm..
